# P05.11. Time, touch, and compassion: effects on autonomic nervous system and well-being

**DOI:** 10.1186/1472-6882-12-S1-P371

**Published:** 2012-06-12

**Authors:** K Kemper, H Shaltout, J Tooze, E Rosenberger

**Affiliations:** 1 Wake Forest University Health Sciences, Winston-Salem, USA

## Purpose

Compassion is critical for complementary and conventional care. This study tested the feasibility of delivering two doses of time (10 and 20 minutes) and two strategies (tactile and non-tactile) for a practitioner to non-verbally communicate compassion (NVCC) to subjects blind to the interventions.

## Methods

Healthy volunteers were informed that the study was testing the effects of time and touch on the autonomic nervous system. Each subject underwent 5 sequential study periods in one study session: (1) Warm-up; (2) Control - with the practitioner while both read neutral material; (3) Rest; (4) Intervention - with practitioner meditating on lovingkindness toward the subject; and (5) Rest. Subjects were randomized to receive one of four interventions: a) 10 minutes tactile; b) 20 minutes tactile; c) 10 minutes non-tactile; or d) 20 minutes non-tactile. During all NVCC interventions, the practitioner meditated on lovingkindness toward the subject. For tactile interventions, the practitioner touched subjects on arms, legs, and hands; for non-tactile interventions, the practitioner pretended to read. Subjects were monitored continuously for autonomic activity. Subjects completed visual analog scales (VAS) for well-being, including relaxation and peacefulness, at warm-up; post-control; immediately post-intervention; and after the post-intervention rest.

## Results

The 20 subjects’ mean age was 24.3 ± 4 years; 16 were women. The practitioner maintained a meditative state during all interventions as reflected in lower RR, and subjects remained blind to the practitioner’s meditative activity. Overall, interventions significantly decreased HR and BP (p<0.01); although other changes did not reach statistical significance, they were in the expected direction, with generally greater effects for the tactile than non-tactile strategies and for 20 minute than 10 minute doses.

## Conclusion

Two strategies are feasible for blinding subjects to non-verbal communication of compassion; even with blinding, non-verbal communication of compassion affects subjects’ autonomic nervous system. Replication is desirable in larger samples.

